# COVID-19 Pandemic: Knowledge and Perceptions of the Public and Healthcare Professionals

**DOI:** 10.7759/cureus.8144

**Published:** 2020-05-15

**Authors:** Priyanka A Parikh, Binoy V Shah, Ajay G Phatak, Amruta C Vadnerkar, Shraddha Uttekar, Naveen Thacker, Somashekhar M Nimbalkar

**Affiliations:** 1 Department of Pediatrics, Pramukhswami Medical College, Karamsad, IND; 2 Central Research Services, Bhaikaka University, Karamsad, IND; 3 Department of Public Health, Child Health Foundation, Gandhidham, IND; 4 Department of Public Health, International Pediatric Association, Gandhidham, IND; 5 Department of Pediatrics, Deep Children Hospital, Gandhidham, IND

**Keywords:** sars-cov-2 (severe acute respiratory syndrome coronavirus -2), covid 19, healthcare provider, public health and safety, knowledge mapping, infection control measures, covid-19 india, lockdown

## Abstract

Background and objective

The recent pandemic due to the novel severe acute respiratory syndrome coronavirus 2 (SARS-CoV-2) has become a major concern for the people and governments across the world due to its impact on individuals as well as on public health. The infectiousness and the quick spread across the world make it an important event in everyone’s life, often evoking fear. Our study aims at assessing the overall knowledge and perceptions, and identifying the trusted sources of information for both the general public and healthcare personnel.

Materials and methods

This is a questionnaire-based survey taken by a total of 1,246 respondents, out of which 744 belonged to the healthcare personnel and 502 were laypersons/general public. There were two different questionnaires for both groups. The questions were framed using information from the World Health Organization (WHO), UpToDate, Indian Council of Medical Research (ICMR), Center for Disease Control (CDC), National Institute of Health (NIH), and New England Journal of Medicine (NEJM) website resources. The questions assessed awareness, attitude, and possible practices towards ensuring safety for themselves as well as breaking the chain of transmission. A convenient sampling method was used for data collection. Descriptive statistics [mean(SD), frequency(%)] were used to portray the characteristics of the participants as well as their awareness, sources of information, attitudes, and practices related to SARS-CoV-2.

Results

The majority (94.3%) of the respondents were Indians. About 80% of the healthcare professionals and 82% of the general public were worried about being infected. Various websites such as ICMR, WHO, CDC, etc., were a major source of information for the healthcare professional while the general public relied on television. Almost 98% of healthcare professionals and 97% of the general public, respectively, identified ‘Difficulty in breathing” as the main symptom. More than 90% of the respondents in both groups knew and practiced different precautionary measures. A minority of the respondents (28.9% of healthcare professionals and 26.5% of the general public) knew that there was no known cure yet. Almost all respondents from both the groups agreed on seeking medical help if breathing difficulty is involved and self-quarantine if required.

Conclusion

Most healthcare professionals and the general public that we surveyed were well informed about SARS-CoV-2 and have been taking adequate measures in preventing the spread of the same. There is a high trust of the public in the government. There are common trusted sources of information and these need to be optimally utilized to spread accurate information.

## Introduction

In December 2019, the 2019 novel coronavirus disease (COVID-19) caused by novel severe acute respiratory syndrome coronavirus 2 (SARS-CoV-2) emerged in China, followed by a rapid spread all over the world. On March 11, 2020, the World Health Organization (WHO) raised its pandemic alert. As of April 11, 2020, COVID-19 had caused over 95,269 deaths in 189 countries and overseas territories or communities [1].

In a connected world, fake news and rumor-mongering are common due to a surge in the use of the internet and social media. A confused comprehension in an emerging communicable disease of which even the experts have inadequate knowledge can lead to fear and chaos, even excessive panic, which has the probability to aggravate the disease epidemic [2]. During the SARS epidemic from 2002 to 2004, there were misconceptions and hence excessive panic in the general public concerning SARS. This led them to be resistant to comply with suggested preventive measures such as avoiding public transportation, going to a hospital when sick, etc. This contributed to the rapid spread of SARS and resulted in a more serious epidemic situation [3]. A similar experience occurred during the Ebola outbreak in 2009 in Africa. These experiences underscore the vital role of engaging with the general public and healthcare professionals and the importance of monitoring their perception of disease epidemic control, which may affect the compliance of community to the precautionary strategies. Understanding related factors affecting and influencing people to undertake precautionary behavior may also help decision-makers take appropriate measures to promote individual or community health. Hence, it is crucial to understand people’s risk perception and identify their trusted sources of information to effectively communicate and frame key messages in response to the emerging disease [4].

Since it is the novel coronavirus, its epidemiological features are not well known and new studies and publications will take anywhere from a month to a year making it important to know and understand the level of knowledge and preparedness of the healthcare personnel in terms of the managing the virus affected patients. Today healthcare professionals managing COVID-19 across the world are in an unprecedented situation, having to make tough decisions and working under extreme pressures. Decisions include equitable distribution of scant resources among the needy patients, balancing their own physical and mental healthcare needs along with those of the patients, aligning their desire and duty to patients with those to family and friends, and providing care for all unwell patients with constrained or inadequate resources. This may cause some to experience moral distress or mental health problems [5].

Effective communication is a priority in WHO’s COVID-19 roadmap; accurate and salient messages will enhance trust and enable the public to make informed choices based on recommendations [6].

As the outbreak intensified, social media has taken on new and increased importance with the large-scale implementation of social distancing, quarantine measures, and lockdown of complete cities. Social media platforms have become a way to enable homebound people to survive isolation and seek help, co-ordinate donations, entertain, and socialize with each other.

Social media platforms arguably support the conditions necessary for attitude change by exposing individuals to correct, accurate, health-promoting messages from healthcare professionals

In order to investigate community responses to SARS-CoV-2, we conducted this online survey among the general public and healthcare professionals to identify awareness of SARS-CoV-2 (perceived burden and risk), trusted sources of information, awareness of preventative measures and support for governmental policies and trust in authority to handle SARS-CoV-2 outbreak and put forward policy recommendations in case of similar future conditions.

## Materials and methods

We performed a cross-sectional survey of a convenient sample of respondents. The ethical approval for the study was taken from the Institutional Ethics Committee - 2, HM Patel Centre for Medical Care and Education, Karamsad via letter IEC/ HMPCMCE/ 2019 / Ex. 07/ dated March 23, 2020. All participants were above 18 years of age conveniently selected from the public at large by reaching out to the general public and healthcare professionals by the authors. The participants were largely from India. The consent of the participants was taken at the beginning of the survey. Two different self-administered questionnaires were used. The one for non-medical personnel (general public) is shown in Table 1, while the one for medical and paramedical personnel is shown in Table 2.

**Table 1:** COVID-19 (for non-medical personnel) question list

COVID-19 (for non-medical personnel) question list	
1	Country
2	Age
3	Sex
4	Are you aware of COVID-19 or coronavirus?
5	Are you worried that you can get infected?
6	If no, why?
7	Where do you get the information regarding coronavirus or COVID-19 from?
8	Do you go to any specific websites?
9	If yes, name of the website
10	What are the symptoms of the disease that you know?
11	How does the disease spread?
12	How can you prevent the spread and protect yourself?
13	Who should wear a mask?
14	Do you wash your hands more frequently now?
15	Are you aware of the technique of handwashing and use of sanitizer?
16	How many times do you wash your hands?
17	Do you avoid social gatherings or events?
18	Have you cancelled a personal trip?
19	If you are suffering from any of the symptoms but not having difficulty in breathing what will you do?
20	If you have fever, cough and shortness of breath what should you do?
21	Do you think the government of India is taking proper steps to control the spread of the disease?
22	Do you believe that there is a treatment for the disease?
23	Do you believe that there is a vaccine for the disease?
24	Do you take the influenza vaccine every year?
25	Do you have old people at home who take the influenza vaccine?
26	If someone gets infected, for how long can he infect others?
27	If you are exposed to an infected person, how long will it take to show symptoms of the disease?
28	Would you be willing to self-isolate and work from home for 7 to 14 days if needed?
29	Is your organization giving you the provision of working from home?
30	What steps do you take to protect yourself?

**Table 2:** COVID-19 (for medical and paramedical personnel) question list WHO, World Health Organization

COVID-19 (for medical and paramedical personnel) question list	
1	Country
2	Profession
3	If other, specify
4	Age
5	Sex
6	Are you worried that you can be infected with coronavirus?
7	If no, why?
8	Where do you get the information regarding coronavirus or COVID-19 from?
9	Do you go to any specific websites?
10	If yes, name of the website
11	Have you read articles published in scientific journals with respect to COVID-19?
12	Have you attended online or in-person any lectures organized by college, IMA or other professional organization?
13	Have you listened to talks on YouTube by WHO or other experts?
14	What source of information do you trust?
15	If website, specify the website
16	If any other source, specify
17	What are the symptoms of the disease that you know?
18	How does the disease spread?
19	How can you prevent the spread and protect yourself?
20	Are you avoiding social/public gathering?
21	If yes, since when?
22	Who should wear a mask?
23	Should you wash your hands before wearing and after removing a mask?
24	Do you wash your hands more frequently now and are you aware of WHO guidelines for handwashing?
25	How many times do you wash your hands?
26	How many steps are there for hand washing as recommended in WHO guidelines for hand hygiene?
27	When should you wash your hands?
28	If you are suffering from any of the symptoms but not having difficulty in breathing what will you do?
29	If you have fever, cough and shortness of breath what should you do?
30	Do you think the government is taking proper steps to control the spread of the disease?
31	Do you believe that there is a treatment for the disease?
32	Do you believe that there is a vaccine for the disease?
33	Do you take influenza vaccine every year?
34	Do you have old people at home who take influenza vaccine?
35	If someone gets infected, for how long can he infect others?
36	If you are exposed to an infected person, how long will it take to show symptoms of the disease?
37	Is COVID-19 same as SARS (severe acute respiratory syndrome)?
38	Have you previously managed SARS (severe acute respiratory syndrome) or other epidemics that cause respiratory issues?
39	For how long the virus will survive on
40	Can you get the infection from your pet (cats and dogs)?
41	Have you cancelled a personal trip?
42	Would you be willing to self-isolate and work from home for 7 to 14 days if needed?
43	Is your organization giving you the provision of working from home?
44	Are you taking hydroxychloroquine?
45	Do you trust the task force of the ICMR on COVID-19?
46	Which stage of the pandemic is India in?
47	What steps do you take to protect yourself?

The questions were framed using information from the WHO, UpToDate, Indian Council of Medical Research (ICMR), Center for Disease Control (CDC), National Institute of Health (NIH), and New England Journal of Medicine (NEJM) website resources as updated till March 19, 2020. They were validated consensually by experts from the Department of Pediatrics, Pulmonary Medicine, Public Health, and General Internal Medicine. The COVID-19 questions for healthcare professionals, i.e., medical and paramedical personnel were applicable to consultants, residents, interns, medical students, physiotherapists, physiotherapy students, nurses, nursing students, dentists, etc. The questionnaire was administered in English with the help of Google forms, which is a cloud-based data management tool used for designing and developing web-based questionnaires and available free. A link to the online surveys was sent out to them via e-mails and different social media platforms, namely WhatsApp, Facebook, LinkedIn, and Instagram messages, hence without any geographical barrier. The data collection was started on the March 23, 2020 and was continued up till March 27, 2020 midnight. The dates are important as on 22 March there was a self-imposed Janata Curfew in response to Prime Minister of India’s call while from the midnight of March 24, 2020, there was a nationwide lockdown across India. The data was automatically collected in the form of a google sheet and the collected data was being exported automatically to google sheets (similar to Microsoft Excel).

Descriptive statistics [mean (SD), frequency (%)] were used to portray the characteristics of the participants as well as their awareness, sources of information, attitudes, and practices related to SARS-CoV-2. Due to large sample sizes in the healthcare professional group as well as the general public group, exploratory visual comparisons were presented without typical statistical tests of significance.

## Results

A total of 744 health and allied professionals and 502 persons from people at large consented and completed the survey. A majority (94.3%) of the participants were Indian residents with insignificant responses from outside India. It is presumed that the majority of the respondents are of Indian residents but the possibility of a handful of them being non-Indians cannot be ruled out because we did not collect demographic data. A comparison of awareness about SARS-CoV-2 between the general public and healthcare professionals is shown in Table 3.

**Table 3:** Awareness about SARS-CoV-2

Awareness about SARS-CoV-2				
	Healthcare professionals	%	General public at large	%
Respondents	744		502	
Country of residence (India)	725	97.4%	450	89.6%
Gender (female)	374	50.3%	219	43.6%
Age (year) - mean	29.55		32.16	
Age (year) - SD	12.53		13.32	
Worried about getting infected	594	80%	410	82%
Major sources of information				
Healthcare professional	483	64.9%	201	40%
Scientific journals	304	40.9%	NA	
Websites	529	71%	147	29.3%
Television	481	64.6%	408	81.3%
Newspapers/magazines	428	57.5%	348	69.3%
Social networks	5	1%	393	78.3%
Identified “difficulty in breathing” as main symptom	727	98%	486	97%
Precautionary measures				
Hand washing	732	98.4%	497	99%
Wearing mask	611	82.1%	344	68.5%
Using sanitizer	704	94.6%	474	94.4%
Avoid public gatherings	721	96.9%	495	98.6%
Maintaining 1-meter distance	697	93.7%	478	95.2%
Avoid touching nose, eyes, mouth	685	92.1%	467	93%
Covering mouth while coughing and sneezing	721	96.9%	482	96%
Self-quarantine when needed	675	90.7%	471	93.8%
Avoid public transport	714	96%	480	95.6%
Knew there is no curative treatment	215	28.9%	133	26.5%
Knew there is no vaccine	438	58.9%	299	59.6%
Infected person can spread it up to 14 days	534	71.8%	315	62.7%
One can be asymptomatic up to 15 days after infection	702	94.3%	456	91%
Who should wear medical mask?				
Healthcare workers	719	96.6%	471	93.8%
Persons with respiratory symptoms	711	95.6%	456	90.8%
Healthy people to protect themselves	303	40.7%	253	50.4%
Person who is coughing/sneezing	652	87.6%	442	88%
Will ask for COVID-19 test for symptoms without difficulty in breathing	306	41%	246	49%

The gender distribution was equal in the healthcare professionals group, whereas it was more male-dominated in the general public group (49.7% vs 56.4% males). The respondents were younger in the healthcare professionals group as compared to the general public group [mean (SD) age: 29.55 (12.53) vs 32.16 (13.32) years].

The majority of the participants from the healthcare professionals group [594 (80%)] and the general public group [410 (82%)] were worried about getting SARS-CoV-2 infection. Those who were not worried expressed justified reasons (mainly precautions) for their attitude. Online resources, television, peer group discussions, and scientific literature constituted the main sources of information in the healthcare professionals group, whereas television, social networking sites, and newspapers/magazines constituted the main sources of information in the general population group. Participants in both groups reported WHO and official Indian Government websites (ICMR, Ministry of Health and Family Welfare (MOHFW)) as the most trusted online resources.

Most of the healthcare professionals reported that they had accessed videos by WHO/other sources [514 (69%)], read scientific articles [407 (54.7%)], and attended online lectures [242 (32.5%)] related to SARS-CoV-2.

Most healthcare professionals [727(98%)] as well as the general public [486(97%)] identified “difficulty in breathing” as the main symptom of SARS-CoV-2 infection along with cough and fever. Respondents from both the groups were aware of precautionary measures such as hand washing/sanitizer, wearing masks, social distancing, covering mouth while sneezing, and self-quarantine. Majority of the participants (62.7% in the general public and 71.8% in healthcare professionals) were aware of the infection period and the asymptomatic period (91% in the general public and 94.3% in healthcare professionals), but there appeared to be some confusion regarding curative treatment and vaccine availability in both the groups. Most participants rightly endorsed medical masks for healthcare workers, symptomatic patients, and persons who are coughing/sneezing. However, an appreciable proportion of healthcare professionals [303(40.7%)], as well as respondents from the general public [253(50.4%)], wrongly endorsed medical masks for healthy persons to protect themselves. 

Most healthcare professionals [648(87.1%)] expressed their trust in the ICMR task force on SARS-CoV-2. Similar feelings were echoed by the general public [426(85%)] in trusting the current government. 

Half of the general public respondents showed eagerness for the SARS-CoV-2 test without difficulty in breathing. A similar trend was observed among health professionals. Almost all respondents from the general public (98%) and the healthcare professionals (100%) endorsed seeking medical help if the breathing difficulty was involved.

Slightly more healthcare professionals reported regular influenza vaccination as compared to the general public [175(23.5%) vs 76(15.1%)]. Almost all the respondents agreed for self-isolation if needed. The majority of the respondents reported that they were washing the hands more frequently and knew the correct way of handwashing.

## Discussion

We present here a study of the awareness of SARS-CoV-2 among healthcare professionals and the general public with a comparison of many features among them. It is heartening to note that the knowledge with respect to SARS-CoV-2 is relatively high among the respondents.

There are, however, various limitations of the study and these are inherent due to the circumstances in which this survey was done. The study was begun on March 23, 2020, one day after Janata Curfew in India as requested by the Prime Minister and one day before the lockdown on March 24, 2020 [7]. The survey was filled during the days of the lockdown when the respondents had a lot of time on their hands and were probably active on social media as well as watching the television news. Hence, it is quite relevant that many individuals have their information from these two sources, making it important to ensure that accurate information through verified channels and healthcare professionals are presented and broadcasted to the people. This also points towards the importance of the right people being active on social media so that they can communicate the scientifically validated information to the masses.

The curfew and the lockdown ensured that the seriousness of the disease was impressed upon by the highest offices in the country, which is reflected in people taking good precautionary measures to protect themselves from the disease as well as break the chain of transmission. The cases in India have hence not risen to a very high number as rapidly as expected/projected, which also probably indicates that the message was well conveyed and well perceived. As this is a survey that was filled remotely, we need to be cautious in drawing strong conclusions.

Another limitation of the study is that the questionnaire was in the form of google forms and the language of conduct was English. This implies that the people who did not have access to the internet and were not literate were unable to be a part of this survey. But as the source of information for all the general public remains similar (television is ubiquitous in India), we can infer that they would have a similar response. We base this inference as the main sources of information of the public at large were newspapers, television, and WhatsApp despite having access to websites and other online sources. In villages, often the literate readout regional newspapers and news received on mobiles to the rest of the family/friends to ensure dissemination of information.

It is now known that the basic reproductive number (R0) of coronavirus is more in healthcare professionals as compared to the lay public and hence the relative indifference or "no worries" approach of healthcare professionals towards getting infected by SARS-CoV-2 is a concern. In the scenario where adequate personal protective equipment (PPE) may not be available to the healthcare facilities in India due to increased global demand, it is important that healthcare workers know their risk for being infected. In a recent study in Mumbai, 79% of the healthcare professionals were aware of the various PPE required with only 54.5% of them being aware of isolation procedures needed for SARS-CoV-2 infected patients [8]. The numbers for paramedical staff were also lower. India imports raw materials for PPE production from China and South Korea. Due to the shortage of materials and low rate of supply, the availability has taken a massive hit resulting in an acute shortage in the market. It is highly likely that many healthcare professionals will not use appropriate PPE, will get infected, and further spread infections to patients [9-11]. The Bhilwara cohort in Rajasthan is an example of how a healthcare professional needs to protect against infection since he/she is likely to transmit it to others [12]. Another example in Mumbai is Saifee hospital, which was shut down due to an infected healthcare professional who continued to work and passed on the infection to many during the asymptomatic phase. The SARS-CoV-2 disease presents a unique organism that can be spread for at least five days before developing symptoms and up to 37 days after presentation [13,14]. Given its high infectivity, it is a recipe for disaster if healthcare personnel gets it. We have not collected demographic information from the participants and hence it is possible that many of them work in situations where they may not anticipate getting infected. The previous few months have shown how surgeons, orthopedicians, dentists, etc., who typically do not deal with infectious diseases are getting infected by coronavirus [15,16]. In this scenario, it is worrying that only 80% of healthcare professionals were worried while the public was slightly more worried (82%).

The difference in the source of information for healthcare professionals and the general public is stark when we compare information garnered through social media. Social media at 78.3% is the second-highest source for the general public, while the healthcare professionals give it a measly 1%. Since social media is prone to fake news, it is heartening that healthcare professionals are not learning from it. However, the reliance of the general public on social media indicates that healthcare professionals, professional organizations, and government officers need to invest a significant proportion of their time and resources to be active on social media to disseminate correct news. The shots heard round the world rapid-response network is an example that needs to be followed [17]. In another example, we have Dr. Roberto Burioni who has successfully given accurate data on social media. If more healthcare professionals were to enrich social media, it would be a useful platform for the public [18,19]. While many government officials are active on Twitter in India, the platform that is commonly used in India is WhatsApp, Telegram, Instagram, and TikTok and these are dynamic and keep changing. WhatsApp in the middle of this pandemic reduced the forwarding to just one person for a message that had been forwarded five times from the previous number of forwarding to five people (which was unlimited initially) [20]. It indicates the importance of this platform across the world for the spreading of messages. The healthcare professionals rated scientific journals at just about 40.9%. It may be due to the low availability of high-quality evidence or poor access that many healthcare professionals in India have to scientific journals, which are mostly published out of developed countries [21]. In a pandemic situation, this disparity in access can be catastrophic and hence most journals have provided open access to all coronavirus-related publications. Healthcare professionals accessed websites such as WHO, Medscape, MOHFW, CDC, Worldometers, covid19.com, ICMR, UpToDate, and PubMed, for reliable information, which is an indicator of their faith in health organizations across the world. Interestingly though at a low 29.3%, much of the general public accessed similar websites such as WHO, MOHFW, CDC, and ICMR. At the time that the survey was administered, online webinars via zoom or other applications were just beginning in India to educate clinicians searching for answers. This is not reflected in our current study due to many of the responses being filled before the same or the respondents not being part of these audiences. The study authors have attended many of these meetings conducted by the Indian Academy of Pediatrics, etc., and this information is made available via email or WhatsApp messages. In a changing world, both healthcare professionals and the general public need to have reliable and accurate sources of information.

The severity of illness was well identified by all who were surveyed as being difficulty in breathing. Another heartening aspect was that precautionary measures were well known to both the groups of participants with appropriate hand washing techniques, avoidance of public gatherings, and covering of the mouth while coughing and sneezing as the top three precautionary measures. During the first week of March in India, all the telephone and cellular caller tunes were changed to advisories of how to prevent coronavirus disease and when to seek medical help, which included the above messages apart from appeals on television, etc [22].

There was less knowledge related to treatment and vaccine among both healthcare professionals and the general public, which was a disappointing finding for healthcare professionals as they were expected to be aware of this. The same could be said of the knowledge of the infectivity period and duration of being asymptomatic after infection. There was a good knowledge of the usage of masks among the general public and healthcare professionals except for the usage of medical masks for healthy people to protect themselves. The ICMR and other bodies have issued guidelines on the usage of masks and this seems to have been disseminated widely [23]. There was also a low insistence on the need for testing those without respiratory difficulty. In a scenario where testing resources are limited, this is an appropriate response but since it is possible to have the infection without respiratory difficulty, especially early on, this disinterest in getting tested, especially in healthcare personnel is worrisome when there is enough evidence of spread from asymptomatic and mildly symptomatic persons. It is also likely that this response may be due to the fact during the time that this questionnaire was administered, the total cases rose from 400+ to about 800+ and the testing strategy of ICMR was limited to those with contact or travel to SARS-CoV-2-affected areas [24].

Since writing this manuscript, except for a single source event of a religious gathering in Delhi, which caused the doubling of cases to increase from about seven days to 4.1 days, it is reasonable to conclude that adequate knowledge exists among the general public. We can only hope that this would be enough to ensure that lockdown to reduce transmission and flatten the curve will be successful [25-28].

## Conclusions

The COVID-19 pandemic has affected the world in various ways. The deficiency of information, the need for accurate information, and the rapidity of its dissemination are important, as this pandemic requires the cooperation of entire populations. The rapid survey that we conducted had a good response and we show that healthcare professionals and the general public were quite well informed about the coronavirus. They are aware of the measures needed to be taken to reduce the spread of the disease. The knowledge present allows the authors to speculate that the lockdown in India would be effective. The public receives a large amount of information from social media such as WhatsApp and the medical fraternity and government need to develop strategies to ensure that accurate information needs to spread in these fora. The public awareness is quite high and it is important that the knowledge of communication channels be known and be kept at the topmost priority throughout the pandemic.
